# A Mobile Phone–Based Gait Assessment App for the Elderly: Development and Evaluation

**DOI:** 10.2196/14453

**Published:** 2020-05-26

**Authors:** Runting Zhong, Pei-Luen Patrick Rau

**Affiliations:** 1 School of Business Jiangnan University Wuxi China; 2 Department of Industrial Engineering Tsinghua University Beijing China

**Keywords:** aged, gait, mHealth, telemedicine, falls prevention

## Abstract

**Background:**

Gait disorders are common among older adults. With an increase in the use of technology among older adults, a mobile phone app provides a solution for older adults to self-monitor their gait quality in daily life.

**Objective:**

This study aimed to develop a gait-monitoring mobile phone app (Pocket Gait) and evaluate its acceptability and usability among potential older users.

**Methods:**

The app was developed to allow older adults to track their gait quality, including step frequency, acceleration root mean square (RMS), step regularity, step symmetry, and step variability. We recruited a total of 148 community-dwelling older adults aged 60 years and older from two cities in China: Beijing and Chongqing. They walked in three ways (single task, dual task, and fast walking) using a smartphone with the gait-monitoring app installed and completed an acceptability and usability survey after the walk test. User acceptability was measured by a questionnaire including four quantitative measures: perceived ease of use, perceived usefulness, ease of learning, and intention to use. Usability was measured using the System Usability Scale (SUS). Interviews were conducted with participants to collect open-ended feedback questions.

**Results:**

Task type had a significant effect on all gait parameters, namely, step frequency, RMS, step variability, step regularity, and step symmetry (all *P* values <.001). Age had a significant effect on step frequency (*P*=.01), and region had a significant effect on step regularity (*P*=.04). The acceptability of the gait-monitoring app was positive among older adults. Participants identified the usability of the system with an overall score of 59.7 (SD 10.7) out of 100. Older adults from Beijing scored significantly higher SUS compared with older adults from Chongqing (*P*<.001). The age of older adults was significantly associated with their SUS score (*P*=.048). Older adults identified improvements such as a larger font size, inclusion of reference values for gait parameters, and inclusion of heart rate and blood pressure monitoring.

**Conclusions:**

This mobile phone app is a health management tool for older adults to self-manage their gait quality and prevent adverse outcomes. In the future, it will be important to take factors such as age and region into consideration while designing a mobile phone–based gait assessment app. The feedback of the participants would help to design more elderly-friendly products.

## Introduction

### Background

With an increasing aging population, 11.9% of the Chinese population was 65 years and older in 2018 [1]. Approximately 28% to 35% of older adults aged 65 years and older fall each year [2]. Gait assessment is useful for older adults because they are vulnerable to frailty or fall risk. For example, gait speed is a well-known indicator of functional ability [3]; stride-to-stride variability might be a predictor of a future fall [4] or frailty [5,6]. Moreover, gait characteristics have been found to be associated with cognitive impairment [7]. Early detection of the decline of gait parameters may help older adults adopt timely interventions, such as gait training, to maintain health and improve their quality of life.

Regarding the influence of age on gait parameters, studies seem to reveal conflicting results. According to Menz et al [8], older adults exhibited a more conservative gait pattern compared with younger adults, characterized by reduced velocity, shorter step length, reduced acceleration root mean square (RMS), and increased step timing variability. Koss et al [9] derived multiple gait parameters from an iPod to predict age-related gait changes and found that younger adults had a more variable, less predictable, and more symmetric gait pattern compared with older adults.

Dual tasks (DTs, walking while conducting a secondary task) are usually applied in gait studies to amplify the effects as they require additional cognitive resources and are common for older adults in daily life [10,11]. A demanding task (eg, fast walking [FW] and the addition of a cognitive distraction) might enhance the sensitivity and specificity of frailty prediction and is recommended for frailty assessment using gait analysis [12]. Smith et al [11] found that a cognitive task was more challenging for older adults than a motor task when they were performing the timed up and go (TUG) test. DT gait may result in decreased walking speed [13] and step frequency [13], increased step time variability [10] and stride time variability [10], and double support time variability [10].

Older adults are increasingly using smartphones and mobile apps. Providing health-related apps for gait assessment would help older adults improve their health outcomes and reduce the burden of care. Several studies have tested validity and reliability of gait analysis using a smartphone [14-17]. Nishiguchi et al [18] found that the reliability and validity of a smartphone in measuring step variability, autocorrelation, and acceleration RMS was comparable with an external accelerometer. Manor et al [15] created an iPhone app for assessment of normal and DT walking and found that the app was valid and reliable in measuring stride timing, compared with the gold standard—instrumented GAITRite mat (CIR Systems, PA) [15]. In a previous study, we established reference gait parameters (walking speed, step frequency, RMS, amplitude variability, step variability, step regularity, and step symmetry) of nonfrail and prefrail older adults under single tasks (STs) and DTs [13]. The study found that prefrail older adults showed significantly decreased speed, mediolateral RMS, vertical RMS, anteroposterior RMS, vertical amplitude variability, and vertical step regularity compared with nonfrail older adults [13]. However, there may be a bias about whether older adults will accept using such technologies in their daily life. To overcome such challenges, it is necessary to involve older adults in the evaluation of such apps to improve design and acceptability.

Existing studies examined older adults’ acceptance of a health-related app. A study by Liu et al [19] reveals the possibility to predict users’ technology acceptance with socioeconomic variables [19]. According to a survey conducted in Hong Kong, 24.10% (995/4129) smartphone or tablet owners had a health app. Tracking physical activity (67.0%, 667/995) and logging health records (43.0%, 428/995) were the most common functions of the health apps. Overall, a younger age, higher education, and higher household income were associated with having health apps. Engaging in moderate physical activity (≥1 day/week, compared with physical inactivity) and having a history of chronic diseases were also associated with having health apps. The study showed a lower prevalence of use of information and communication technologies (ICTs) in respondents with lower education and income in the most developed Chinese city. This could be seen as a confirmation of the *inverse information law*, which suggests that those most in need have less use of services, and hence, receive less benefits from advancements in health-related ICTs [20]. Inspired by this phenomenon, it is necessary to investigate the acceptability among older adults from different socioeconomic positions to contextually inform specific policies to promote the app. In this study, we used two cities Beijing and Chongqing with different socioeconomic levels in China as examples. Beijing is in North China, with an average gross domestic product (GDP) of 128,994 Chinese Yuan (CNY), whereas Chongqing is in West China, with an average GDP of 63,442 CNY [21].

### Objectives

The study had two aims: (1) develop a gait-monitoring mobile phone app (Pocket Gait) and evaluate its acceptability and usability among potential older users, and (2) conduct gait assessment using the app and examine the influence of age group, task type, and region. The main contribution of this study was that we developed low-cost mobile phone apps using an Android smartphone (vivo Z1, Android operating system version 8.1, VIVO Technology Co, China) compared with the gold standard—instrumented GAITRite mat. The app discussed in this paper could assist with daily gait assessment. In addition, the acceptability and usability results could provide design recommendations to promote use of the app among Chinese older adults.

## Methods

### Gait Assessment App Development

#### Key Design Considerations

**Figure 1:**
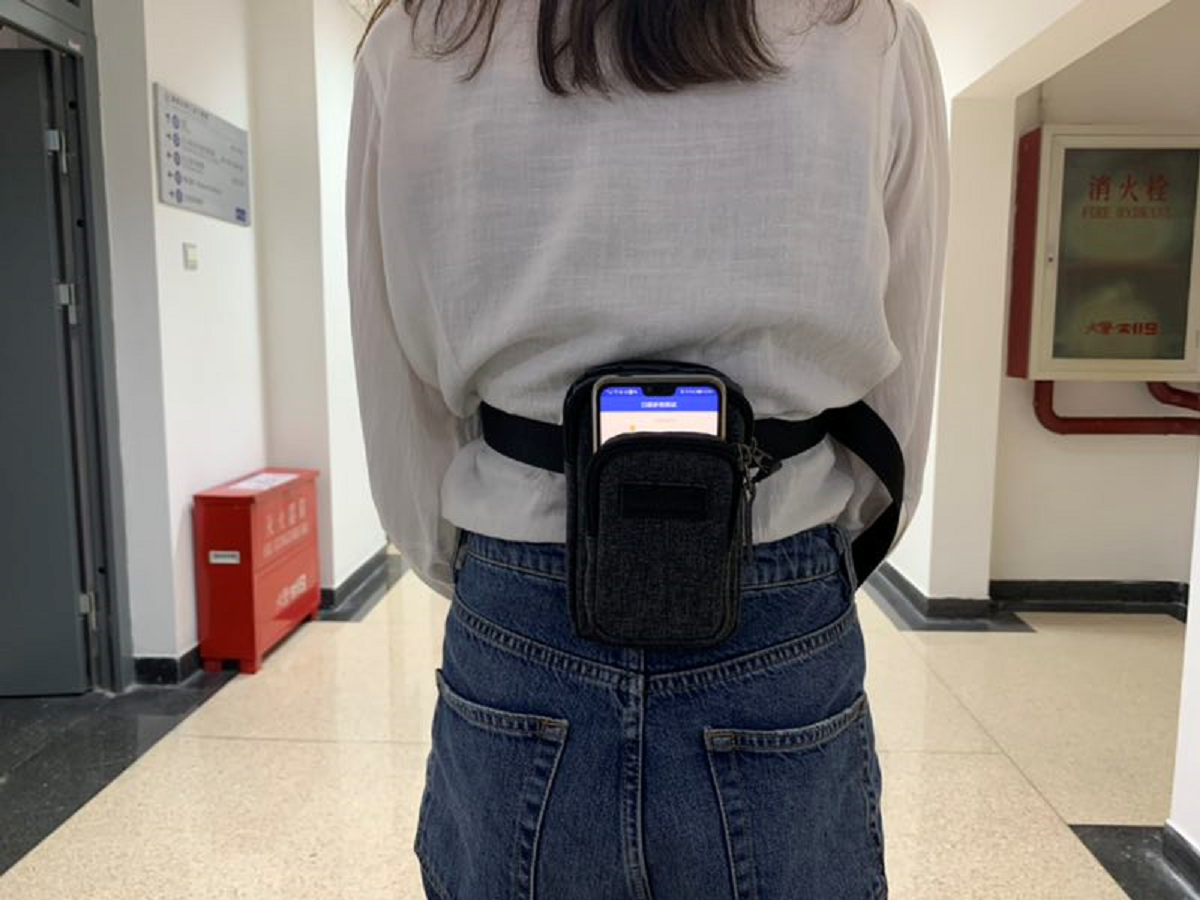
The smartphone was placed in a pocket near the third lumbar spine vertebra (L3) of the lower back. The screen of the smartphone faced outward.

*Pocket Gait* was designed to achieve the goal of monitoring gait quality in daily life. As an accelerometer is commonly embedded in smartphones, it could be used to collect gait data. A previous study illustrated the importance of tracking and giving feedback [22]. Older adults suggested that the data display should enable users to understand the results better. For example, a gait analysis report is required to explain the results with graphs, conclusions, and medical advice [22]. The design of the gait assessment app was based on this idea. The key design requirements are listed as follows:

- Gait test: Users wore a smartphone on the third lumbar spine vertebra (L3) region of the back [23], as shown in Figure 1. When the user was walking, the smartphone would start collecting acceleration data of the three axes.
- Viewing graph: The interface displayed the vertical acceleration pattern to reveal the periodicity of walking. A researcher could also check if the data were being collected properly.
- Viewing report: The interface displayed the critical gait parameters of straight walking (step frequency, step intensity [RMS], step regularity, step symmetry, and step variability).
- Send: Users sent the gait data to researchers via email. Meanwhile, the raw data were stored locally in the smartphone.

#### System Architecture

**Figure 2:**
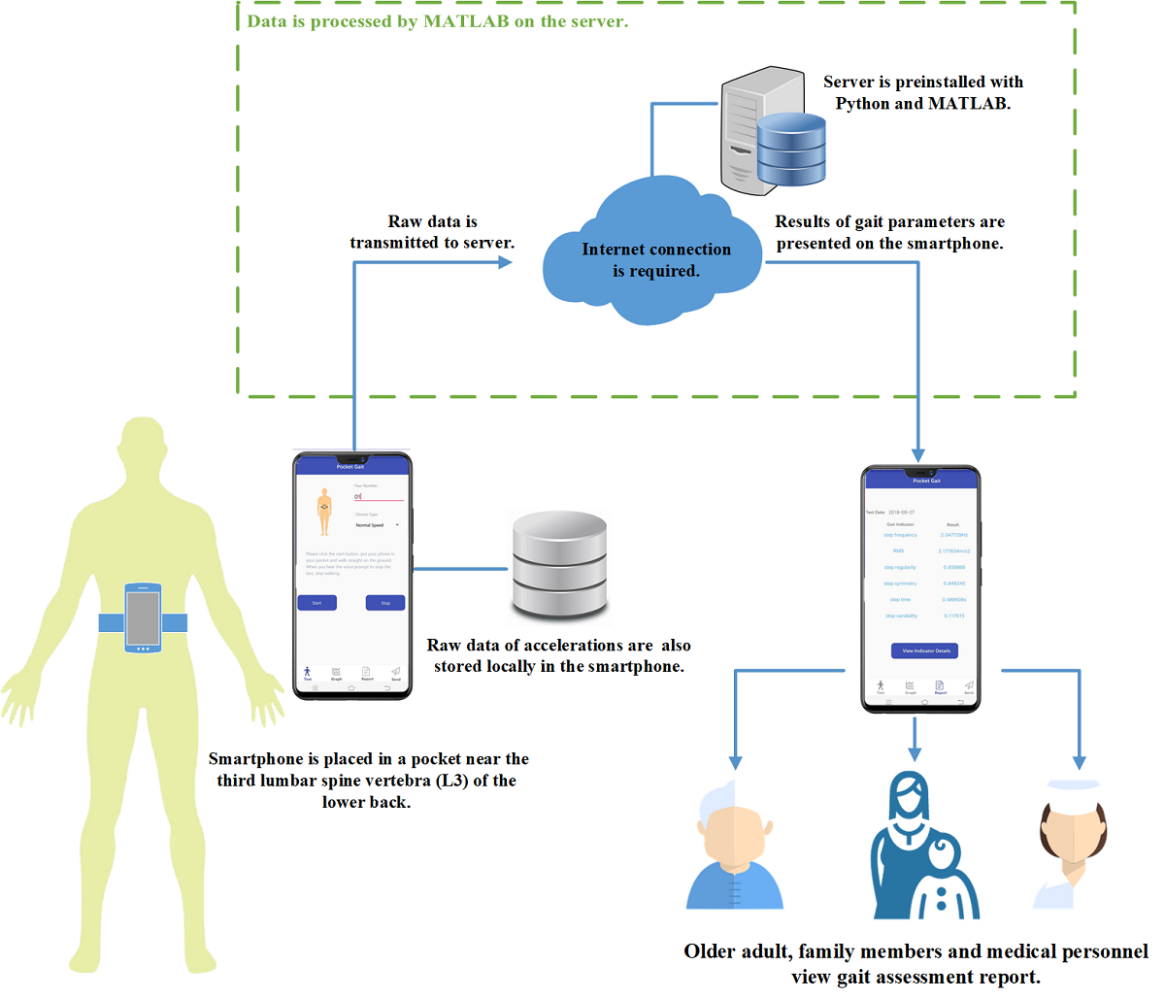
System architecture of the Pocket Gait app.

The gait assessment app was developed on an Android smartphone to allow gait data collection and presentation of the results. The system architecture was set up as follows:

- The Android smartphone served as a client, detecting motion (acceleration) when the user was walking and sending these data to the server.
- The server was installed on a computer with Python (Python Software Foundation) and MATLAB (Mathworks, Natick, MA) preinstalled, receiving and processing gait data from the smartphone.
- The smartphone received the gait assessment results and displayed them on the screen.
- Figure 2 illustrates the system architecture of Pocket Gait.

#### System Development

The gait-monitoring app was initially developed with the overall goal to monitor gait quality, including step frequency, acceleration RMS, step regularity, step symmetry, and step variability. The algorithm for the gait parameters was based on a previous study and developed using a self-designed MATLAB program [13]. A detailed description of the algorithm can be found in Multimedia Appendix 1.

The mobile phone app was developed using Android Studio, as Android is also the most popular operating system among Chinese smartphone users, with almost 80% of the share as of July 2017 [24]. The sampling rate of acceleration measurement for the smartphone was set at the highest mode listed in the specifications for an Android smartphone, which is SENSOR_DELAY_FASTEST [25]. The actual sampling rate was around 40 Hz. The initial 5 seconds were not included in data collection to avoid the influence of the acceleration process. When the user pressed the *Start* button, the app would collect the data from the 5th second to 35th second. In other words, the app would collect 30 seconds of the walking data. There were voice instructions about how to walk when the user pressed the *Start* button. In addition, there were voice instructions reminding the user to stop walking at the end of the gait assessment. The app had three walking types: ST, cognitive DT, and FW. For ST, the participant should walk at normal speed. For DT, the participant should walk while serially subtracting 3 from a 3-digit number randomly given by the experimenter, stating the answers out loud. For FW, the participant should walk as fast as possible.

When pressing the *Graph* button in the navigation bar, the interface would display the vertical acceleration pattern to reveal the periodicity of walking.

**Figure 3:**
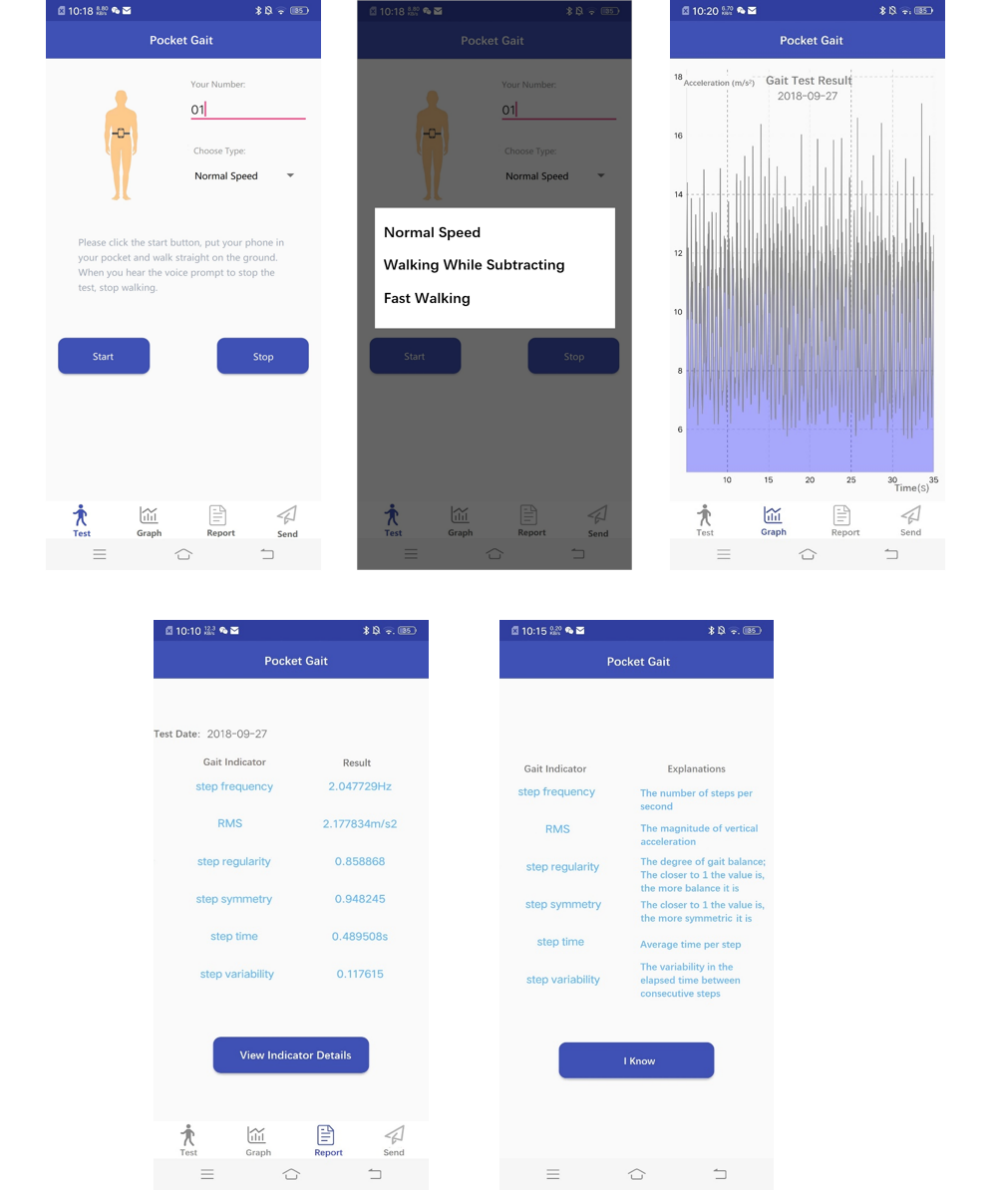
Screenshots of the Pocket Gait app: the gait test page, the choosing walking type page, the viewing graph page, the report page, and the explanation page.

When pressing the *Report* button in the navigation bar, the collected acceleration data were automatically uploaded to a remote server via Wi-Fi. The gait indicators step frequency, acceleration RMS, step variability, step regularity, and step symmetry were then calculated by the MATLAB engine on the remote server and displayed on a smartphone screen, as shown in Figure 3.

### Empirical Data Collection and User Evaluation

To evaluate the proposed prototype, we conducted user evaluations in a corridor over a distance of about 40 meters, collecting evaluations from participants after using the app.

#### Participant Recruitment

In October 2018, a total of 148 older adults were recruited from universities and nearby communities in Beijing (n=70) and Chongqing (n=78). The older adults were recruited through recruitment flyers, word of mouth, and social media. The inclusion criteria were (1) age ≥60 years, (2) living independently in the community, and (3) being able to walk independently without an assistive device for at least 40 meters. Participants were excluded if they had any musculoskeletal or neurological disease, or painful conditions, that could affect gait. Tsinghua University gave ethical approval for the study. Each participant was asked to provide written informed consent before participation. Each participant was given 80 CNY after he or she completed the experiment.

#### Study Design

The experiment used a 2×3×3 mixed design. The 3 independent variables were the region (Beijing or Chongqing), task type (ST, DT, or FW) and age group (60–69, 70–79, or 80–89 years). The between-subject variables were region and age group of the participants. The within-subject variable was task type.

#### Procedure

First, using structured questionnaires, the researchers recorded the participants’ background information, including age, sex, height, weight, fall history in the past 6 months, education, smartphone experience, and internet experience. Fall history was determined by asking the question “Have you ever fallen unintentionally in the past six months?” Smartphone experience was determined by asking the question “Are you using a smartphone?” Internet experience was determined by asking the question “Do you use the internet?” For each participant, the Activity-Specific Balance Confidence Scale (ABC) [26] was used for measuring the fear of falling, the TUG test [27] was performed for measuring mobility, and the one leg stance (OLS) test [28] was performed for measuring balance.

Second, the participants were asked to take gait tests under the following three task types using the app: ST, DT, and FW conditions. The participants were initially asked to walk at a comfortable speed. Then, the participants were asked to walk at a comfortable speed while serially subtracting 3 from a 3-digit number randomly given by the experimenter, stating the answers out loud. Finally, the participants were asked to walk as fast as possible. All the participants wore comfortable footwear. The smartphone was placed in a waist-worn pocket, located close to the L3 region.

Third, participants completed the acceptability and usability survey with the researchers’ help. The survey comprised 3 sections. The first section included 10 items measured on a 5-point Likert scale, with responses ranging from strongly agree to strongly disagree. The questionnaire used to measure acceptance was adopted from a study by Zhou et al [29]. There were four quantitative measures of acceptance (Multimedia Appendix 2): perceived ease of use, perceived usefulness, ease of learning, and intention to use. The second section included usability testing with the System Usability Scale (SUS). The SUS measures the usability of a product and consists of 10 items which are evaluated on a 5-point Likert scale ranging from 1 for *strongly disagree* to 5 for *strongly agree*. The results were distributed on a specific scale ranging from 0 for *worst imaginable* to 100 for *best imaginable* [30].

Finally, the participants were asked the following questions during the interviews: (1) What features of this app do you like? (2) What features of this app do you dislike? and (3) What do you think are the potential improvements for this app?

#### Statistical Analysis

All statistical analyses were performed using IBM SPSS for Windows (version 22.0). Regarding the demographics and functional performance of the participants, normality was assessed using the Kolmogorov-Smirnov test. Independent *t* tests were used for the measures that were distributed normally. The Mann-Whitney *U* tests were used for the measures that were not distributed normally. Pearson chi-square tests were used to analyze the difference in categorical variables (sex, fall history, education, smartphone owner, and internet user) between participants from Beijing and Chongqing.

The gait parameters collected by the smartphone were analyzed using repeated measures analysis of variance (ANOVA). The gait data were assessed using the Mauchly test of sphericity. If sphericity was violated, the Greenhouse-Geisser correction was made. If the task type or the interaction effects were significant, post hoc tests were performed using the least significant difference (LSD). The level of significance was set at *P*<.05.

The user evaluation of acceptability and usability of the app was analyzed using descriptive statistics and ANOVA. We examined the differences in responses by participant characteristics (age and region). The interview data about participants’ recommendations were analyzed using content analysis. The open-ended responses were analyzed using magnitude coding, a process that quantifies participants’ answers, highlighting the most frequent comments.

## Results

### Participants’ Characteristics

The participants’ characteristics are presented in Table 1. Most of the participants were female (108/148, 73.0%). The mean age of the participants was 69.8 (SD 7.0) years, ranging from 60 to 87 years. We compared demographics and functional performance of participants from Beijing and Chongqing. The participants from Beijing had greater values for height, weight, education, being a smartphone owner, and for being an internet user than participants from Chongqing (*P*<.05). Participants from Beijing also performed better in ABC, TUG, and OLS (*P*<.001).

**Table 1:** Demographics and functional performance of participants in this study (N=148).

Variables			All (N=148)		Beijing (n=70)		Chongqing (n=78)		*P* value
Age (years), mean (SD)			69.8 (7.0)		70.5 (7.7)		69.2 (6.2)		.27
**Sex, n**									**.06**
	Male	40		24		16			
	Female	108		46		62			
Height (cm), mean (SD)			159.4 (8.2)		162.2 (7.4)		156.7 (8.1)		<.001
Weight (kg), mean (SD)			62.5 (12.8)		65.1 (15.5)		60.5 (9.2)		.02
**Fall history in the past 6 months^a^, n**									**.85**
	Yes	37		18		19			
	No	111		52		59			
**Education, n**									**<.001**
	Primary	38		1		37			
	Middle school	54		20		34			
	High school or technical secondary school	28		22		6			
	College or junior college	28		27		1			
**Smartphone owner^b^, n**									**<.001**
	Yes	109		64		45			
	No	39		6		33			
**Internet user^c^, n**									**<.001**
	Yes	89		54		35			
	No	59		16		43			
Activity-Specific Balance Confidence scale (%), mean (SD)			89.7 (10.2)		92.4 (8.8)		87.0 (10.8)		.001
Timed up and go (seconds), mean (SD)			9.5 (2.1)		8.5 (1.4)		10.3 (2.2)		<.001
One leg stance (seconds), mean (SD)			21.2 (9.8)		25.7 (7.2)		17.9 (10.3)		<.001

^a^Fall history was determined by asking the question “Have you ever fallen unintentionally in the past six months?”

^b^Smartphone experience was determined by asking the question “Are you using a smartphone?”

^c^Internet experience was determined by asking the question “Do you use the internet?”

### Gait Assessment

After checking the data collected by the smartphone, the data of 8 participants were excluded because of abnormal collection (missing data). Therefore, the number of participants included for gait analysis was 140. Of the 8 excluded participants, 6 were from Beijing, and 2 were from Chongqing; 5 were in the age group 60 to 69 years, 1 was in the age group 70 to 79 years, and 2 were in the age group 80 to 89 years. In all, 3 of the excluded participants were male, and 5 were female.

#### Step Frequency

Table 2 presents statistics for step frequency. ANOVA indicated that age group (*F_2,134_*=0.45, *P*=.50) and task type (*F_1.72,230.43_*=204.16, *P*<.001) had significant effects on step frequency. The step frequency was 2.07, 2.01, and 1.95 Hz for the age groups 60 to 69 years, 70 to 79 years, and 80 to 89 years, respectively. The post hoc analysis showed the step frequency of participants in the age group 60 to 69 years was significantly higher than that of participants in the other age groups.

Step frequency was 1.85, 2.18, and 1.99 Hz for task types DT, FW, and ST, respectively. The post hoc analysis showed that step frequencies in DT, FW, and ST were significantly different from each other (*P* values<.001). The lowest step frequency was observed in DT, whereas the highest step frequency was observed in ST.

**Table 2:** Statistics for step frequency.

Variables		Descriptive analysis, mean (95% CI)	Analysis of variance			
			*F* (*df*)		*P* value	
**Region**				**0.45 (1)**		**.50**
	Beijing (n=64)	2.00 (1.95-2.04)				
	Chongqing (n=76)	2.02 (1.97-2.07)				
**Age group (years)**				**4.61 (2)**		**.01^a^**
	60-69 (n=79)	2.07 (2.03-2.10)				
	70-79 (n=43)	2.01 (1.96-2.05)				
	80-89 (n=18)	1.95 (1.88-2.03)				
**Task type**				**204.16 (1.72)**		**<.001^a^**
	Dual task	1.85 (1.81-1.89)				
	Fast walking	2.18 (2.15-2.22)				
	Single task	1.99 (1.96-2.02)				

^a^Significant at .05 level.

#### Acceleration Root Mean Square

Table 3 presents statistics for RMS. Task type (*F_1.78,237.84_*=302.94, *P*<.001) and region×task type (*F_1.78,237.84_*=6.15, *P*=.004) were demonstrated to have significant effects on RMS.

For participants from Beijing, RMS in DT, FW, and ST was 1.97, 3.14, and 2.26 m/s^2^, respectively. The post hoc analysis showed that RMS in DT, FW, and ST were significantly different from each other (*P* values <.001). RMS was highest in FW and lowest in DT.

For participants from Chongqing, RMS in DT, FW, and ST was 2.05, 3.06, and 2.36 m/s^2^, respectively. The post hoc analysis showed RMS in DT, FW, and ST were significantly different from each other (*P* values <.001). RMS was highest in FW and lowest in DT.

**Table 3:** Statistics for root mean square.

Variables		Descriptive analysis, mean (95% CI)	Analysis of variance	
			*F* (*df*)	*P* value
**Region**			**0.11 (1)**	**.75**
	Beijing	2.46 (2.31-2.60)		
	Chongqing	2.49 (2.33-2.65)		
**Age group (years)**			**2.10 (2)**	**.13**
	60-69	2.53 (2.41-2.64)		
	70-79	2.60 (2.44-2.76)		
	80-89	2.29 (2.04-2.55)		
**Task type**			**302.94 (1.78)**	**<.001^a^**
	DT^b^	1.97 (1.85-2.09)		
	FW^c^	3.14 (3.01-3.27)		
	ST^d^	2.31 (2.20-2.42)		
**Region×task type**			**6.15 (1.78)**	**.004^a^**
	DT (Beijing)	1.89 (1.73-2.05)		
	FW (Beijing)	3.22 (3.05-3.40)		
	ST (Beijing)	2.26 (2.11-2.41)		
	DT (Chongqing)	2.05 (1.87-2.23)		
	FW (Chongqing)	3.06 (2.87-3.26)		
	ST (Chongqing)	2.36 (2.20-2.52)		

^a^Significant at .05 level.

^b^DT: dual task.

^c^FW: fast walking.

^d^ST: single task.

#### Step Variability

Table 4 presents statistics for step variability. Task type (*F_1.59,212.89_*=16.77, *P***<**.001) and age×task type (*F_3.18,212.89_*=3.57, *P*=.01) were demonstrated to have significant effects on RMS.

For participants in the age group 60 to 69 years, step variability in DT, FW, and ST was 0.093, 0.13, and 0.093, respectively. Step variability in DT was significantly lower than that in FW (*P*<.001). Step time variability in FW was significantly higher than that in ST (*P*<.001).

For participants in the age group 70 to 79 years, step variability in DT, FW, and ST was 0.105, 0.121, and 0.092, respectively. Step variability in FW was significantly higher than that in ST (*P*<.001).

For participants in the age group 80 to 89 years, step variability in DT, FW, and ST was 0.098, 0.091, and 0.077, respectively. Step variability in DT was significantly higher than that in ST (*P*=.003).

**Table 4:** Statistics for step variability.

Variables		Descriptive analysis, mean (95% CI)	Analysis of variance			
			*F* (*df*)		*P* value	
**Region**				**3.83 (1)**		**.05**
	Beijing	0.091 (0.080-0.10)				
	Chongqing	0.11 (0.095-0.12)				
**Age group (years)**				**1.20 (2)**		**.30**
	60-69	0.10 (0.095-0.11)				
	70-79	0.11 (0.094-0.12)				
	80-89	0.089 (0.069-0.11)				
**Task type**				**16.77 (1.59)**		**<.001^a^**
	DT^b^	0.099 (0.090-0.11)				
	FW^c^	0.11 (0.10-0.12)				
	ST^d^	0.087 (0.079-0.09)6				
**Age group×task type**				**3.57 (3.18)**		**.01^a^**
	60-69 (DT)	0.093 (0.083-0.10)				
	70-79 (DT)	0.11 (0.091-0.12)				
	80-89 (DT)	0.098 (0.077-0.12)				
	60-69 (FW)	0.13 (0.11-0.14)				
	70-79 (FW)	0.12 (0.11-0.14)				
	80-89 (FW)	0.091 (0.065-0.12)				
	60-69 (ST)	0.093 (0.084-0.10)				
	70-79 (ST)	0.092 (0.079-0.11)				
	80-89 (ST)	0.077 (0.056-0.097)				

^a^Significant at .05 level.

^b^DT: dual task.

^c^FW: fast walking.

^d^ST: single task.

#### Step Regularity

Table 5 presents statistics for step regularity. Region (*F_1,134_*=4.51, *P*=.04), task type (*F_1.45,194.66_*=57.30, *P*<.001), and age group×task type (*F_2.91,194.66_*=7.02, *P*<.001) had significant effects on step regularity. Step regularity for participants from Beijing and Chongqing was 0.75 and 0.79, respectively. Participants from Beijing had significantly lower step regularity than participants from Chongqing (*P*=.04).

For participants in the age group 60 to 69 years, step regularity in DT, FW, and ST was 0.76, 0.79, and 0.81, respectively. The post hoc analysis showed that step regularity values in DT, FW, and ST were significantly different from each other (*P* values <.05). Step regularity was highest in ST and lowest in DT.

For participants in the age group 70 to 79 years, step regularity in DT, FW, and ST was 0.71, 0.80, and 0.80, respectively. Step regularity in DT was significantly lower than that in FW (*P*<.001) and in ST (*P*<.001).

For participants in the age group 80 to 89 years, step regularity in DT, FW, and ST was 0.67, 0.83, and 0.79, respectively. Step regularity values in DT, FW, and ST were significantly different from each other (*P* values <.05).

**Table 5:** Statistics for step regularity.

Variables		Descriptive analysis, mean (95% CI)	Analysis of variance			
			*F* (*df*)		*P* value	
**Region**				**4.51 (1)**		**.04^a^**
	Beijing	0.75 (0.73-0.78)				
	Chongqing	0.79 (0.77-0.82)				
**Age group (years)**				**0.69 (2)**		**.50**
	60-69	0.79 (0.77-0.81)				
	70-79	0.77 (0.74-0.80)				
	80-89	0.76 (0.72-0.81)				
**Task type**				**57.30 (1.45)**		**<.001^a^**
	DT^b^	0.72 (0.69-0.74)				
	FW^c^	0.81 (0.79-0.83)				
	ST^d^	0.80 (0.78-0.82)				
**Age group×task type**				**7.02 (2.91)**		**<.001^a^**
	60-69 (DT)	0.76 (0.73-0.79)				
	70-79 (DT)	0.71 (0.68-0.75)				
	80-89 (DT)	0.67 (0.61-0.73)				
	60-69 (FW)	0.79 (0.77-0.82)				
	70-79 (FW)	0.80 (0.77-0.83)				
	80-89 (FW)	0.83 (0.78-0.88)				
	60-69 (ST)	0.81 (0.78-0.83)				
	70-79 (ST)	0.80 (0.77-0.83)				
	80-89 (ST)	0.79 (0.74-0.84)				

^a^Significant at .05 level.

^b^DT: dual task.

^c^FW: fast walking.

^d^ST: single task.

#### Step Symmetry

Table 6 presents the statistics for step symmetry. Task type (*F_1.60,214.51_*=13.52, *P*<.001) had significant effects on step symmetry. Step symmetry in DT, FW, and ST was 0.89, 0.93, and 0.93, respectively. Step symmetry in DT was significantly lower than that in FW (*P*<.001) and in ST (*P*<.001).

**Table 6:** Statistics for step symmetry.

Variables		Descriptive analysis, mean (95% CI)	Analysis of variance			
			*F* (*df*)		*P* value	
**Region**				**2.09 (1)**		**.15**
	Beijing	0.91 (0.88-0.93)				
	Chongqing	0.93 (0.91-0.96)				
**Age group (years)**				**0.67 (2)**		**.51**
	60-69	0.92 (0.90-0.94)				
	70-79	0.91 (0.88-0.93)				
	80-89	0.93 (0.89-0.97)				
**Task type**				**13.52 (1.60)**		**<.001^a^**
	Dual task	0.89 (0.87-0.92)				
	Fast walking	0.93 (0.91-0.95)				
	Single task	0.93 (0.91-0.95)				

^a^Significant at .05 level.

### Acceptability

Overall, the acceptability feedback from users was positive for the four quantitative measures of acceptance (Table 7), indicating that participants acknowledged the perceived ease of use, perceived usefulness, ease of learning, and intention to use.

**Table 7:** Descriptive statistics for the acceptability of the app.

Function	Perceived ease of use, mean (SD)	Perceived usefulness, mean (SD)	Ease of learning, mean (SD)	Intention to use, mean (SD)
Gait test	3.7 (0.6)	3.9 (0.4)	3.7 (0.6)	3.5 (0.8)
Viewing graph	3.6 (0.8)	3.8 (0.5)	3.6 (0.7)	3.6 (0.7)
Viewing report	3.5 (0.7)	3.8 (0.5)	3.6 (0.7)	3.5 (0.8)

### Usability

The data of 2 participants were excluded because of missing data. Therefore, the number of participants included for usability analysis was 146. Participants identified the usability of the system with an overall SUS score of 59.7 (SD 10.7) out of 100. In terms of sex, there was no noticeable difference for the perception of usability. Male participants evaluated the usability of the system with a score of 62.1 (SD 11.6), whereas female participants evaluated the usability with a score of 58.8 (SD 10.3).

Regarding region, there was a significant difference in the SUS score between participants in Beijing and Chongqing (*t_144_*=4.17, *P*<.001), indicating that participants in Beijing (mean 63.4, SD 9.8) had a higher level of satisfaction with the gait assessment app compared with participants in Chongqing (mean 56.3, SD 10.5). A possible reason for this difference is that participants in Beijing have higher education than participants in Chongqing.

Moreover, there was a significant difference in the SUS score between age groups (*F_2,145_*=3.09, *P*=.048). The LSD post hoc revealed that participants between 60 and 69 years of age had a higher level of satisfaction (mean 61.4, SD 10.1) than participants over 80 years of age (mean 55.3, SD 11.7; *P*=.02).

### Attitude Toward Key Features of the App

#### Question 1: What Features of This App Do You Like?

During the interviews, participants reported the app features they liked. Of the 140 participants, 48 (34.3%) thought that the app could help them familiarize themselves with their health conditions, and older adults could benefit from the app both physically and mentally:

You can know the condition of how you walk in your daily life and see the results at a glance. You can plan how to walk in your own life and walking would become a daily fun.

It is good to see the speed and balance during walking. If the result is good, the mood is good.

It is good for health, helping the elderly to train their brain.

In all, 21 of 140 (15.0%) participants considered the app to be convenient to use:

I think the application is convenient.

It is easy to use the application. I will know about the situation at a glance.

It is flexible. You can use it anytime and anywhere as long as you have a mobile phone.

Of the 140 participants, 6 (4.3%) thought that the app gave objective indicators that could not be observed by eye:

I like the authenticity of the app. Slow is slow.

The app reflects the physical condition objectively.

Of the 140 participants, 5 (3.5%) expressed the intention to use the app as an incentive for walking or exercise:

Walking is good. I like walking.

It urges me to take exercise.

The application reflected the whole process of walking, which could help you exercise in a way that suits you.

The application could offer scientific quantitative analysis, which is good for walking.

#### Question 2: What Features of This App Do You Dislike?

Next, participants also reported the app features that they disliked, expressing difficulties of using the app. Of 140 participants, 37 (26.4%) said that there was nothing they did not like about the app. Of 140 participants, 25 (17.9%) complained about the small font size on the display, as one of them mentioned:

The font size of the application is too small to see clearly.

Of 140 participants, 14 (10.0%) older adults thought that using the app was too complicated for them to learn and mentioned that “The system is a little bit too complicated,” but 3 of them showed that they could learn to use the app with the help of others (eg, a teacher or daughter).

In all, 5.7% (8/140) participants complained about the graph, as one of them pointed out:

I don’t understand this graph. It is better to explain which range is right/good and which range is wrong/bad. It is better to have a normal range.

Of 140 participants, 5 (3.6%) thought that the gait indicators in the report were difficult to understand. Some participants thought that the result should be saved and recalled:

It’s very difficult to learn. I remembered it at that time and then I would forget it. I walk if I have to, and I don’t have to look at it. I use mobile phone for the elderly, so I do not know about this.

In all, 3.5% (5/140) participants thought that the functions of the app should be supplemented. One participant mentioned:

It would be better to include blood pressure and heart rate. When exercising, elderly people are required to reach a state of slight sweating. The app could be a reminder of excessive exercise and be used to detect the warning signs of sudden death during sports.

Another participant said:

The function is relatively simple if it simply measures walking. Walking may be good, but the balance system may not be good. It is better to give some guidelines on how to improve the balance system.

Of 140 participants, 3 (2.1%) regarded the device restrictions as disadvantages:

*The application has equipment restrictions.* *It will be better if it could be used in a mobile phone for the elderly.*

Overall, 2 of 140 (1.4%) participants thought it was unnecessary to use the app by giving the following reasons:

I rarely go out.

There is no big change in the pace when walking on the ground. If it is serious, the doctor can diagnose it. If it is not serious, the app will not reflect.

One participant complained that the pocket was too large, while another participant said the following:

The size of the smartphone was too large. It would be better to be as small as a smart bracelet.

These responses from the participants explained the usability problems and reflected future directions for improvement.

#### Question 3: What Are the Potential Improvements for This App?

Finally, participants identified various potential improvements for the app. Of 140 participants, 35 (25.0%) suggested that the font size of the app should be larger, and 37 (26.4%) suggested that the functions of the app should be supplemented. Among them, 11 participants suggested that the app should provide more information on the purpose of gait test:

What is the purpose of each test? Does it inform what problem the body has? What does the value of each gait parameter inform? How to improve if the value is higher or lower?

In all, 13 of 140 (9.3%) participants suggested that the app should provide reference values for gait parameters so that the user knows which position they are in:

Adding the reference range of gait parameters. Tell users which range is preferred.

The results must be accurate. Not only must there be results, but there must also be explanations for the reasons of the results, and some medical help.

Regarding the graph, of 140 participants, 1 (0.7%) indicated:

It would be better for the chart to display a curve and for long-term users to be able to provide the data to the doctor to assess the condition, which would allow to doctor to make informed judgements.

Overall, 2 of 140 (1.4%) participants suggested using distinct colors:

It is better to use differentiated colors. It is clearer at a glance.

Some participants suggested that the app should include other body indicators during walking, such as heart rate (11 participants), blood pressure (5), step count (4), vital capacity (1), and distance (1). Some suggested that the app could add other functions, such as the option for background music (2), road condition prompts (2), other exercise activities (1), bone density test (1), and viewing the results of friends (1)*.*

Of 140 participants, 11 (7.9%) thought that the functions of the app should be simplified:

I hope there is a summary of the result and the app informs me whether the result is good or bad. There could be fewer gait indicators.

In all, 4 of 140 (2.9%) participants preferred other wearing positions than the lower back:

*I hope* *the smart phone could be placed on any part of the body, such as on the chest. Placing the smartphone in the pocket of the suit is more convenient than on the back.*

Of 140 participants, 2 (1.4%) suggested changing the environment when conducting the test:

The test could be conducted in an open environment. It is not as enjoyable to walk in a closed environment as it is outdoors.

One (1/140, 0.7%) participant hoped that the app could be used in a mobile phone for the elderly.

## Discussion

### Principal Findings

We developed a gait-monitoring mobile phone app (Pocket Gait) and assessed the acceptability of the app with older adults. We examined the influence of age group, task type, and region on gait parameters. Gait assessment results revealed that task type had a significant effect on all gait parameters, namely, step frequency, RMS, step variability, step regularity, and step symmetry (all *P* values <.001). Age had a significant effect on step frequency (*P*=.01), and region had a significant effect on step regularity (*P*=.04). The step frequency of participants in the age group 60 to 69 years was significantly higher than that of participants in the other age groups. Participants from Beijing had significantly lower step regularity than participants from Chongqing.

We performed acceptability and usability testing among participants. The acceptability of the gait-monitoring app was positive among older adults for the four quantitative measures of acceptance, namely, the perceived ease of use, perceived usefulness, ease of learning, and intention to use. The usability score was 59.7 out of 100. Further interviews indicated some usability problems. Suggestions to improve the usability of the app are presented in Textbox 1. Basic improvements suggested are that the font size should be larger, and more detailed instructions should be provided. To reduce difficulties of using the app, users should be provided with instructions and training, and informed about the meaning of gait parameters and the use of each test when promoting the mobile phone app.

**Suggestions for improvement.**

*Gait test*

1. The app should inform users about the purpose of each gait test.

2. Inclusion of heart rate and blood pressure monitoring in the app.

3. Use higher volume for the voice instructions.

4. The gait test could be conducted in an open environment.

5. It is more comfortable to walk if background music is added.

6. The smartphone could be placed on any part of the body, such as on the chest.

7. Tell the user what range of the gait parameters is acceptable.

8. Change *test* to *activity* (or other words) to relax the users.

*Viewing graph*

9. For the long term, users can provide the data to a doctor to assess the condition.

10. Save the chart and recall it at any time.

*Viewing report*

11. Use larger font size.

12. Add the reference range for gait parameters and tell users which range is preferred.

13. Present results, as well as explanation for the results, and some medical advice.

14. Tell the user how to improve if the value is higher or lower than the reference value.

### Comparison With Previous Work

Other studies in health-related ICT also applied acceptability and usability testing among older adults. Portz et al [31] developed a mobile phone app for tracking symptoms of heart failure among older adults and tested its acceptability. The study found that older age was associated with a need for assistance to use the app. Vaziri et al’s study [32,33] also found that age was an important factor for the system usability evaluation of an ICT-based fall prevention system iStoppFalls. Younger participants assessed the usability of the system better than older participants [33]. Our study showed that age and region are important factors for the usability assessment of the gait assessment app. Participants that were younger and from Beijing assessed the usability of the app better. During the experiment, we found that there were some difficulties for the older adults aged over 80 years, for example, eye disease prevented them from reading the words on the screen. In addition, 39/148 (26.4%) of the older adults in this study did not have a smartphone, so that they had no idea about the app. Therefore, it will be important to take factors such as age and region into consideration when promoting the mobile phone-based gait assessment app. Older users or users with low socioeconomic status may be disadvantaged in using the app. More instructions and social support from the caregivers or family members are needed to promote using the app among such users.

### Limitations and Future Studies

The study has some limitations. First, the study only included community-dwelling older adults from Beijing and Chongqing. Older adults from other regions were not investigated in this study. Second, the gait assessment was conducted in a corridor, which was different from their daily environment. Third, the researchers were helping the participants complete the usability and acceptability testing. If the participant could not read, the researcher would read aloud each item of acceptability and usability. It is worth noting that Chinese older adults tend to give moderate responses during the user evaluation, that is, they seldom responded *strongly disagree* or *strongly agree*. This could partly explain why the SUS score is marginal.

The gait assessment app could be generalized to other populations. For example, it could be an incentive for exercise for sedentary young people. The app could also be used to monitor long-term changes in patients undergoing rehabilitation (eg, stroke and Parkinson disease). As this study evaluated the gait assessment app from the perspective of community-dwelling older adults, future studies could evaluate the app from the perspective of patients or care givers. Finally, the app could be extended to other mobile platforms (eg, iPhone Operating System).

### Conclusions

Smartphones may serve as useful tools to support the gait assessment of older adults and facilitate aging in place, which is defined as “remaining living in the community, with some level of independence, rather than in residential care” [34]. Our study discussed the development and acceptability of a gait-monitoring mobile phone app (Pocket Gait) among Chinese older adults. The study findings established reference values for gait parameters and provided design recommendations for further improvements of the app.

## Appendix

Multimedia Appendix 1 The algorithm to extract gait parameters.

Multimedia Appendix 2 Questionnaires for acceptability and usability.

## Abbreviations

ABC: Activity-Specific Balance Confidence scale
ANOVA: analysis of variance
CNY: Chinese Yuan
DT: dual task
FW: fast walking
GDP: gross domestic product
ICT: information and communication technology
L3: the third lumbar spine vertebra
LSD: least significant difference
OLS: one leg stance
RMS: root mean square
ST: single task
SUS: System Usability Scale
TUG: timed up and go
