# Supplementary material for: A Mobile Phone–Based Gait Assessment App for the Elderly: Development and Evaluation
Source: JMIR Mhealth Uhealth. 2020 May 26;8(5):e14453. doi: 10.2196/14453 (PMC7284482; doi:10.2196/14453)
Supplement: Multimedia Appendix 1 [file mhealth_v8i5e14453_app1.docx]

Description of the algorithm to derive gait parameters

The recorded signals were processed by a self-designed Matlab program in the remote server. As the raw data were not constantly sampled, we adjusted the sampling rate of the acceleration signal to 100 Hz using interpolation in Matlab. Data were detrended and filtered using a low-pass Butterworth filter with a cut-off frequency of 10 Hz. The **vertical** acceleration signals was processed.

**Step frequency** was processed using the fast Fourier transform (FFT).

**The RMS acceleration** indicates the magnitude of the acceleration. It was calculated using the *rms* function in the signal processing toolbox.

**Step variability** is calculated as the coefficient of variance using the following formula:

$$\left[ \frac{t_{SD}}{t_{MEAN}} \right]\times100\%$$

The positive peak of the acceleration was firstly detected by the *findpeak* function in Matlab. The time interval from the adjacent peak was regarded as the stride time *t*. The step variability was then calculated from the mean *t_MEAN_* and the standard deviation *t_SD_* of the time intervals. Higher value of step variability is worse.

**Step regularity** was calculated as an autocorrelation coefficient. The autocorrelation coefficient was calculated using the *xcorr* function in Matlab. The step regularity in this study is the peak value of the autocorrelation coefficient around a step. Higher value of autocorrelation is better.

**Step symmetry** was calculated as the ratio of step regularity $D_{1}$ to stride regularity $D_{2}$:$D_{1}/D_{2}$ if $D_{2}>D_{1}$; $D_{2}/D_{1}$ if $D_{1}>D_{2}$. Here $D_{1}$ represents step regularity and $D_{2}$ represents stride regularity. Higher value of step symmetry is better.
