# Supplementary material for: A Mobile Phone–Based Gait Assessment App for the Elderly: Development and Evaluation
Source: JMIR Mhealth Uhealth. 2020 May 26;8(5):e14453. doi: 10.2196/14453 (PMC7284482; doi:10.2196/14453)
Supplement: Multimedia Appendix 2 [file mhealth_v8i5e14453_app2.docx]

Appendix 2

# The Questionnaire to Measure Acceptance

| Items | Strongly disagree | Disagree | Neutral | Agree | Strongly agree |
| --- | --- | --- | --- | --- | --- |
|  | 1 | 2 | 3 | 4 | 5 |
| **Perceived ease of use:** |  |  |  |  |  |
| 1. I think it is easy to use ___ on this device. |  |  |  |  |  |
| 1. I think I know how to control ___ on this device. |  |  |  |  |  |
| **Perceived usefulness:** |  |  |  |  |  |
| 1. ___ is useful. |  |  |  |  |  |
| 1. ___ is enjoyable. |  |  |  |  |  |
| 1. ___ can make my life more convenient, comfortable, and effective. |  |  |  |  |  |
| **Ease of learning:** |  |  |  |  |  |
| 1. It is easy to learn ___ on this device. |  |  |  |  |  |
| 1. I want to learn how to use ____. |  |  |  |  |  |
| **Intention to use:** |  |  |  |  |  |
| 1. I am interested in using ___. |  |  |  |  |  |
| 1. If I have this device, I intend to use ___. |  |  |  |  |  |
| 1. If I have this device, I will frequently use___. |  |  |  |  |  |

Note: The blank line was replaced with the function name, namely, gait test, viewing graph and viewing report.

# SUS = System Usability Scale

| **Items** | Strongly disagree | Disagree | Neutral | Agree | Strongly agree |
| --- | --- | --- | --- | --- | --- |
|  | 1 | 2 | 3 | 4 | 5 |
| 1. I think that I would like to use this system frequently |  |  |  |  |  |
| 1. I found the system unnecessarily complex |  |  |  |  |  |
| 1. I thought the system was easy to use |  |  |  |  |  |
| 1. I think that I would need the support of a technical person to be able to use this system |  |  |  |  |  |
| 1. I found the various functions in this system were well integrated |  |  |  |  |  |
| 1. I thought there was too much inconsistency in this system |  |  |  |  |  |
| 1. I would imagine that most people would learn to use this system very quickly |  |  |  |  |  |
| 1. I found the system very cumbersome to use |  |  |  |  |  |
| 1. I felt very confident using the system |  |  |  |  |  |
| 1. I needed to learn more information before I could get going with this system |  |  |  |  |  |
